# New insights about pilus formation in gut-adapted *Lactobacillus rhamnosus* GG from the crystal structure of the SpaA backbone-pilin subunit

**DOI:** 10.1038/srep28664

**Published:** 2016-06-28

**Authors:** Priyanka Chaurasia, Shivendra Pratap, Ingemar von Ossowski, Airi Palva, Vengadesan Krishnan

**Affiliations:** 1 Regional Centre for Biotechnology, NCR Biotech Science Cluster, Faridabad—121 001, India; 2 Department of Biotechnology, Manipal University, Karnataka, 576104, India; 3 Department of Veterinary Biosciences, University of Helsinki, Helsinki, Finland

## Abstract

Thus far, all solved structures of pilin-proteins comprising sortase-assembled pili are from pathogenic genera and species. Here, we present the first crystal structure of a pilin subunit (SpaA) from a non-pathogen host (*Lactobacillus rhamnosus* GG). SpaA consists of two tandem CnaB-type domains, each with an isopeptide bond and E-box motif. Intriguingly, while the isopeptide bond in the N-terminal domain forms between lysine and asparagine, the one in the C-terminal domain atypically involves aspartate. We also solved crystal structures of mutant proteins where residues implicated in forming isopeptide bonds were replaced. Expectedly, the E-box-substituted E139A mutant lacks an isopeptide bond in the N-terminal domain. However, the C-terminal E269A substitution gave two structures; one of both domains with their isopeptide bonds present, and another of only the N-terminal domain, but with an unformed isopeptide bond and significant conformational changes. This latter crystal structure has never been observed for any other Gram-positive pilin. Notably, the C-terminal isopeptide bond still forms in D295N-substituted SpaA, irrespective of E269 being present or absent. Although E-box mutations affect SpaA proteolytic and thermal stability, a cumulative effect perturbing normal pilus polymerization was unobserved. A model showing the polymerized arrangement of SpaA within the SpaCBA pilus is proposed.

Among bacteria, certain Gram-positive genera and species are known to display long proteinaceous structures on their outer cell surface. These protrusions (called sortase-dependent pili or fimbriae) are typically built up from protein subunits known as pilins. The genes required for assembling these pili are organized tandemly in the genome as a fimbrial operon and encode for two or three types of pilins (major and ancillary) and one or more pilus-specific (C-type) sortase enzymes^1^,^2^. Here, the sortase-catalyzed assembly of protein subunits yields a covalently linked structure consisting of repeating major pilins that form the pilus backbone, along with two ancillary pilins, a large-sized one for adhesion located at the pilus tip and a much smaller one for cell wall attachment positioned at the pilus base. Characteristically, the backbone and basal pilins often possess a pilin motif (YPKN) within the N-terminal region, in which the conserved lysine residue is essential for incorporating these protein subunits into the pilus structure^3^. For pilus polymerization, the C-type sortase makes a cleavage between the threonine and glycine of the LPXTG motif in a major pilin, and then covalently connects the threonine to the lysyl side chain in the pilin motif of an adjoining major pilin. Structural work on the individual Gram-positive pilins first began in 2007, with crystal structures solved for the *Streptococcus agalactiae* GBS52 basal pilin^4^ and *Streptococcus pyogenes* Spy0128 major pilin^5^. Since then, several other pilin structures have been reported^6^. These pilins are generally comprised of two types of domains, which are variants of immunoglobulin folds known as CnaA and CnaB, and often contain intramolecular isopeptide bonds^6^,^7^,^8^,^9^.

Because the Gram-positive pili have mainly been characterized in pathogens, these surface appendages are seen as key virulence factors for promoting host-cell adhesion and pathogenesis^1^,^2^,^10^. This particular view of sortase-dependent pili has changed somewhat with their detection in less harmful bacteria^11^,^12^,^13^, where they are now regarded as niche-adaptation factors. As the first reported host of “non-pathogenic-derived” pili^11^,^12^, *Lactobacillus rhamnosus* GG is a gut-adapted commensal lactic acid bacterium (LAB) and one of many clinically investigated probiotic strains for human use in the prevention and treatment of certain gastric ailments^14^. The “GG” notation for the *L. rhamnosus* GG strain is derived from the surnames of Barry Goldin and Sherwood Gorbach, who had originally isolated it from healthy human intestine in 1983^14^. However, the molecular mechanisms and actions that are responsible for the health-promoting effects of this strain remain to be defined. Key to this outcome is the strong adherence ability of *L. rhamnosus* GG cells, which seems to help prolong their transient (allochthonous) colonization and survival in the gastrointestinal (GI) tract. Here, the surface piliation uncovered in *L. rhamnosus* GG appears to play a central role.

As with other Gram-positive bacteria, sortase-dependent piliation in *L. rhamnosus* GG (called SpaCBA) is assembled from the ~30 kDa SpaA (backbone) (Fig. 1a), ~20 kDa SpaB (basal), and ~90 kDa SpaC (tip-located) pilins. Their corresponding genes, along with a C-type sortase (SrtC1) gene, are encoded in the fimbrial *spaCBA* operon (i.e., *spaC*–*spaB*–*spaA*–*srtC1*)^11^,^15^,^16^. Incidentally, another pilus operon (called *spaFED*) can be found in the *L. rhamnosus* GG genome, although its expression is constitutively silent^16^, with the corresponding surface piliation (called SpaFED) only producible as a recombinant form^17^. As a function, the *L. rhamnosus* GG SpaCBA pilus is reported to display a number of properties, which includes the adherence to mucus^11^,^18^,^19^, collagen protein^20^, and gut epithelial cells^21^, but as well, the stimulation of biofilm growth^21^ and various host-cell immune responses^21^,^22^,^23^,^24^,^25^. Undoubtedly, this type of surface piliation offers a valuable niche-specific fitness to *L. rhamnosus* GG cells when they occupy the intestinal milieu^15^.

In our present study, we have initiated a structural investigation of the *L. rhamnosus* GG pilin constituents^26^,^27^,^28^, with the overall aim of understanding the structure-function properties of individual pilins in commensal bacteria, and determining any differences from their counterparts in pathogens. As a first report, we now present the structural determination of a backbone-pilin subunit from a gut-benefiting bacterium using X-ray crystallography. Here, the crystal structure of *L. rhamnosus* GG SpaA (hereafter denoted as GG-SpaA to avoid confusion with the corynebacterial SpaA backbone-pilin subunit^29^, which, although likewise named, shares little primary structure similarity) is determined at 1.9 Å resolution, along with its N- and C-terminal domains at 2.0 Å and 1.6 Å resolution, respectively. Two immunoglobulin (Ig)-like subdomains comprise GG-SpaA, each having the conserved residues of a canonical E-box motif and internal isopeptide bonds formed between either lysine and asparagine or lysine and aspartate residues. Our biochemical experiments suggest that the E-box glutamate residue might function in the proteolytic and thermal stability of GG-SpaA. Crystal structures of mutant proteins having single residue replacements (E139A, E269A, and D295N) and an involvement with isopeptide bond formation were also solved, and while the E139A substitution maintains the overall fold (albeit with some minor changes), the E269A change causes different conformational effects over time. Here, the E269A substitution initially has a tendency to destabilize protein folding when isopeptide bonds are absent, but later surprisingly, it retains the overall fold following the formation of intact isopeptide bonds. With E269A mutant protein, we were able to solve the crystal structure of the N-terminal domain, which is notably flexible, and has not been elucidated previously in any other Gram-positive pilins by X-ray crystallography. Based on the remarkable resemblance to a native-like pilus structure in the molecular packing of the GG-SpaA crystal, we also propose a model that provides some insight into how the *L. rhamnosus* GG SpaCBA-pilus backbone is structured.

## Results

### Structural determination of GG-SpaA

**Figure 1:**
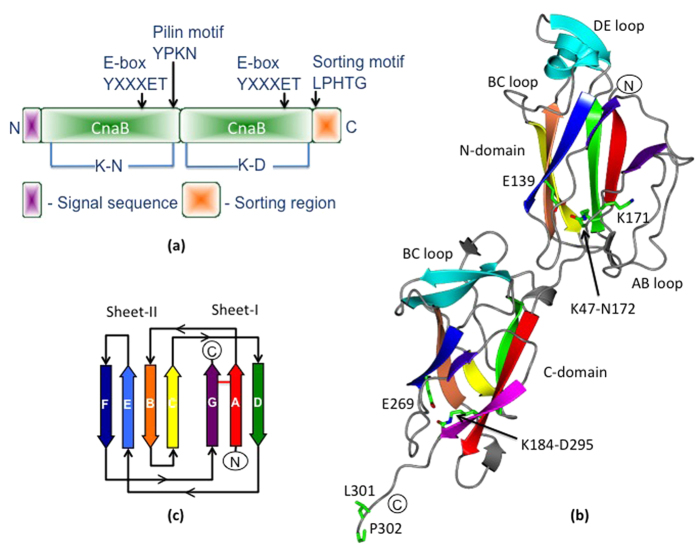
Structure of the GG-SpaA backbone-pilin subunit. (**a**) Schematic representation showing the predicted domain organization of GG-SpaA. GG-SpaA consists of two CnaB-type domains, with regions for the secretion and LPXTG motif sorting signals at the N- and C-terminals, respectively. Arrows indicate positions of the pilin motif and E-box. Residue locations for the lysine-asparagine (K-N) and lysine-aspartate (K-D) isopeptide bonds are shown. (**b**) Ribbon cartoon of the GG-SpaA crystal structure. GG-SpaA consists of two Ig-like domains, with each having a CnaB-type fold. N- and C-terminally located domains are labeled as N-domain and C-domain, respectively. Core β-strands forming the β-sandwich fold are rainbow colored (red to violet) according to the topology diagram (see Fig. 1c). Residues forming isopeptide bonds are shown in sticks. The essential lysine (K171) of the pilin motif at the domain interface and the leucine (L301) and proline (P302) residues of the C-terminally located LPXTG motif are also shown in sticks. Extended loop regions are labeled accordingly. Major β-strands unrelated to the core CnaB fold are shown in cyan. (**c**) Topology diagram depicting the secondary structure of the GG-SpaA CnaB fold. Core β-strands of the β-sandwich fold are sequentially labeled (A to G) starting from the N- to C-terminals and rainbow colored (red to violet). An isopeptide bond between β-strands A and G is indicated by a red horizontal line.

Recombinant SpaA from *L. rhamnosus* GG (GG-SpaA) was cloned and expressed in *E. coli*^19^, and then purified^28^. The GG-SpaA construct lacks the regions for the N-terminal secretion and C-terminal sorting signals (Fig. 1a), but includes the leucine and proline residues of the LPXTG motif, which themselves are followed by the leucine and glutamate residues encoded by the cloning vector and the six histidine residues of the C-terminal His-tag^28^. Crystals of full-length GG-SpaA protein belonging to the monoclinic *C*2 space group diffracted up to 1.9 Å resolution at a synchrotron source (BM14, ESRF) (Table 1). Our initial attempts to solve the structure using molecular replacement or heavy atom derivative techniques were both unsuccessful. As an alternative, GG-SpaA protein was fragmented by limited proteolysis as had been done previously for other backbone pilins^26^,^30^. This produced a 15 kDa-sized fragment that can be crystallized in two different forms. Whereas the tetragonal crystals had diffracted to 2.0 Å and 1.6 Å resolution at home and synchrotron sources, respectively (Table 1), the orthorhombic crystals showed diffraction to 2.0 Å resolution at both sources (Table 1). The structure of the 15 kDa fragment was solved by the single anomalous dispersion method (SAD) using an ytterbium (Yb) derivative of the orthorhombic crystal form. In parallel, we solved the crystal structure of the tetragonal form with an advanced molecular replacement program using search models from distant homologs. As a pruned search template, the C-terminal domain of the *S. agalactiae* GBS52 pilin^4^ yielded an interpretable electron-density map for model building, though it offered less than 20% sequence identity. Refined models for the tetragonal and orthorhombic crystals (residues 176–298) gave R_work_/R_free_ values of 13.9%/15.8% and 21.6%/24.9%, respectively. These models were used to solve the full-length wild-type (WT) GG-SpaA structure (residues 39 to 302), which led to R_work_ and R_free_ values of 17.4% and 21.7%, respectively (Table 1). The WT GG-SpaA model was then used to solve the crystal structures for single residue-substituted mutants (E139A, E269A, and D295N) (Table 2).

### Overall structural topology of GG-SpaA

Crystals for full-length GG-SpaA contain three molecules in the asymmetric unit, where the head of one molecule faces the tail of another. Each GG-SpaA molecule is made up of two Ig-like domains connected by a short linker (Fig. 1b) with an interface area of 641 Å^2^. These two domains are arranged linearly, having an overall length of 93 Å and a total surface area of 13528 Å^2^. The two domains are bent by a hinge angle^31^ (i.e., the angle between the principal ellipsoid axes of the domains) of 152°, in which the N- and C-terminal domains (N-domain and C-domain) consist of residues 39–175 and 176–302, respectively. Seven anti-parallel β-strands arranged in two sheets (I and II) form a β-sandwich core in each domain. The topology of the β-sandwich resembles a CnaB or Ig-rev fold (Fig. 1c)^9^,^32^, with β-strands D, A, and G combining to form sheet I and β-strands C, B, E, and F then forming sheet II (Fig. 1c). The N-domain of GG-SpaA contains two hook-shaped loops (AB and BC) placed at opposite sides. The DE loop forms a β-hairpin that is positioned perpendicular to the β-sandwich core and adjacent to the α-helix of the BC loop. At the C-domain, the AB, DE, and FG loops appear shorter, whereas the BC loop is longer and contains 40 residues folding into a β-hairpin structure that aligns perpendicular to the core β-strands at the domain interface. The C-terminal tail, encompassing residues 299–302 that include a portion of the sorting motif, is seen to extend from the β-sandwich core of the C-domain. The AB loop (residues 50–60), which interacts with the FG loop in the N-domain, was partially disordered in two of the three molecules in the asymmetric unit.

### Formation of intra-domain isopeptide bonds in GG-SpaA

**Figure 2:**
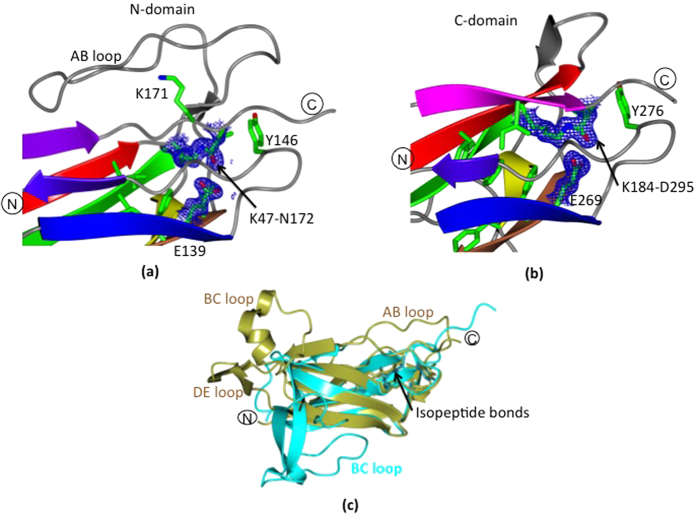
Location of isopeptide bonds in the GG-SpaA structure. (**a**) The isopeptide bond in the N-domain forms between K47 and N172, and also involves the catalytic glutamate at position 139. Residues for isopeptide bond formation (K47, N172, and E139), adjacent hydrophobic residues, and the essential lysine of the pilin motif (K171) are shown in sticks. The electron density (2Fo-Fc) map is contoured at 1.5 σ. (**b**) Isopeptide bond formation in the C-domain involves K184, D295, and catalytic E269. Residues for the isopeptide bond and nearby hydrophobic residues are shown in sticks. The electron density (2Fo-Fc) map is contoured at 1.5σ. (**c**) Superposition of the N- and C-domains (gold and cyan, respectively) is depicted. Location of isopeptide bonds is indicated by an arrow. Neighboring hydrophobic residues are shown in sticks. Major loop perturbations are labeled (AB, BC, and DE).

The electron density map clearly shows continuous density for two intra-domain isopeptide bonds, one for each domain (Fig. 2a,b). Formation of the isopeptide bond (K-N) in the N-domain occurs between Lys47 at the end of β-strand A and Asn172 at the end β-strand G, which corresponds to the pilin motif found near the domain interface (Fig. 2a). A potentially catalytic glutamate residue (Glu139) from β-strand E is in the vicinity and forms a hydrogen bond with the O moiety of the isopeptide bond, which is in the *trans* configuration. The unpaired O of the Glu139 carboxyl group also exhibits a hydrogen-bonding interaction with the O moiety of the isopeptide bond via a nearby water molecule. Moreover, the Tyr146, Leu45, Leu73, Val76, Phe78, Leu115, and Pro170 residues gather together to form a hydrophobic cluster around the N-domain isopeptide bond.

On the other hand, isopeptide bond formation in the C-domain is between Lys184 from β-strand A and Asp295 from β-strand G, and involves a proximal catalytic Glu269 in a hydrophobic pocket made by Tyr276, Phe198, Ile293, Phe182, Phe245, Phe253, Ala196, and Leu193 (Fig. 2b). This isopeptide bond also adopts a *trans* configuration and its O moiety forms a single hydrogen bond with the carboxyl group of Glu269, which is located in β-strand E. The unpaired O of the Glu269 carboxyl group forms a hydrogen bond with a nearby main-chain NH group of Ala279, which is located in β-strand F in the vicinity of the C-terminal sorting motif.

It is worthy of mention that the isopeptide bond involving the side-chain carboxyl group of aspartate and the ε-amino group of lysine (K-D) is uncharacteristic of most Gram-positive pilins, but yet is found occasionally^33^,^34^. In GG-SpaA, Asp295 is located five residues upstream of the sortase recognition motif (i.e., LPHTG). More typically, the equivalent position 5–8 residues from the LPXTG sorting motif is occupied by an asparagine. Nonetheless, K-D isopeptide bonding has already been observed structurally in the middle domains of a tip-pilin (Spy0125 C-terminal region; CTR) and a fibronectin-binding surface adhesin (FbaB) from *S. pyogenes*^33^,^34^. Previous studies have shown that K-N and K-D isopeptide bond formation follows a similar mechanism^35^,^36^, although, in comparison, K-D isopeptide bonds are generated more slowly and will lead to the loss of a molecule of water instead of ammonia. Here, various interactions from the surrounding amino acid environment probably dictate whether isopeptide bond formation involves either an aspartate or asparagine residue^35^. However, more relevantly, these two isopeptide bonds in GG-SpaA have an important structural role by helping enhance protein stability that then favors resistance to proteases.

As depicted in Fig. 2c, superposition of the N- and C-domains of GG-SpaA (RMSD of 2 Å for 86 aligned Cα positions at 16% sequence identity) reveals that there is a similar location and orientation for the residues forming the isopeptide bonds, including proximal negatively charged residues and some hydrophobic ones (Phe78:198, Leu73:193, and Tyr146:276). Moreover, the *trans* configuration of the isopeptide bond and its single hydrogen-bonding pattern with a proximal glutamate residue is also well-conserved between the N- and C-domains. As similarly found with other Gram-positive pilins, the residues from different β-strands that participate in the isopeptide bond formation are found near the domain boundaries at the C-terminal end.

### Metal ion coordination in the C-domain of GG-SpaA

**Figure 3:**
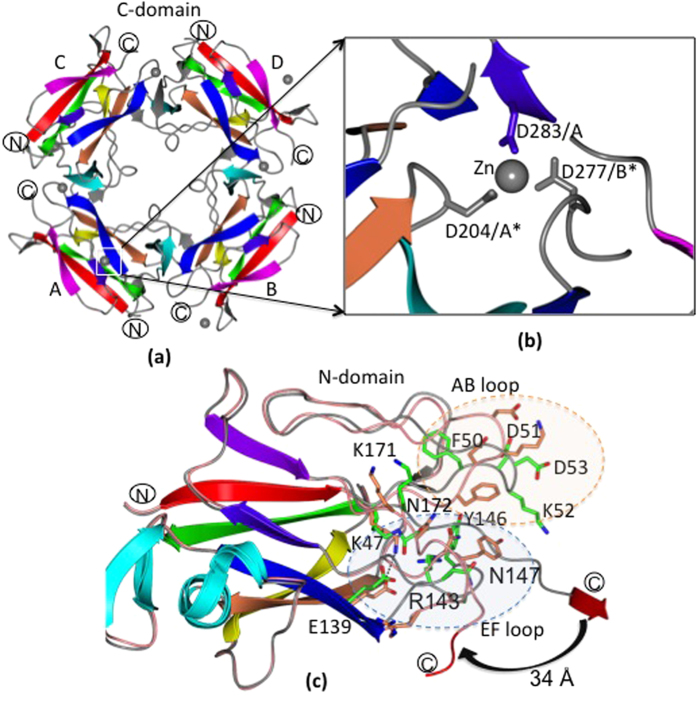
Structure of C- and N-domains of GG-SpaA derived from limited proteolysis and E269A mutant protein, respectively. (**a**) Four copies of the C-domain GG-SpaA are in the asymmetric unit of the orthorhombic crystal form. Core β-strands of the β-sandwich are rainbow colored as in Fig. 1c. Metal ions are depicted as gray spheres. (**b**) Enlarged view of one metal ion coordinated by aspartates from self (D283) and two crystal symmetry mates (D204* and D277*). (**c**) N-domain of GG-SpaA obtained from E269A mutant protein. An isopeptide bond is not formed between K47 and N172, despite the presence of a glutamate at position 139 in the N-domain. While K47 is engaged in hydrogen bonding with E139, N172 is pointing away from K47. The core β-sandwich is rainbow colored as in Fig. 1c, but the loops and critical residues (sticks) are shown in pink for highlighting their conformational changes in comparison with that of full-length WT (in gray and green). Major changes in the AB and EF loops are indicated by an oval (dashed lines) in pink and blue, respectively. Movement of the C-terminal linker region (residues 170–180) is shown by an arrow (black).

The structure of a 15 kDa fragment of GG-SpaA that had been generated by limited proteolysis is identical to that of the C-domain of full-length GG-SpaA (see preceding section for details). However, the electron density for the residues belonging to the extended C-terminal tail was not observed, with proteolytic removal as the likely reason. Intriguingly, six metal ions are associated with the C-domain when the structure was solved using orthorhombic crystals (Fig. 3a). Mainly owing to the crystals having been grown in the presence of 20 mM zinc sulphate, these metal ions were modeled putatively as zinc. Here, these metal ions can be seen at the surface between the molecules in the asymmetric unit stabilizing molecular packing in the crystal (Fig. 3a). Metal ions are primarily coordinated by aspartates (Fig. 3b), but glutamate, lysine, and water from asymmetric and symmetric molecules also seem to participate. Among known categories of zinc-binding sites in proteins^37^,^38^, the metal ion coordination environment in GG-SpaA can be classified as an “interface type”, based on the incomplete coordination spheres, weak electron densities for the coordinated water molecules, and the location of the metal sites. In the lanthanide derivative obtained by co-crystallization for SAD phasing, these metal ion-binding sites are mostly occupied by ytterbium ions.

### Conformational changes in the N-domain of GG-SpaA

Prior to obtaining crystals of full-length E269A-substituted GG-SpaA, this mutant protein had fortuitously degraded to only the N-domain during crystallization (see Methods). Up to now, the crystal structure of an N-terminal domain from a Gram-positive pilin has never been determined before by X-ray crystallography, largely because of its rather flexible nature. Yet, for these particular crystals of the E269A mutant, all N-domain residues were easily built into the electron density map, with the exception of Thr55-Ile57 in the AB loop. Here, the crystal structure of the N-domain exhibited a major conformational change at the domain interface region, but with the overall fold still retained (RMSD of 1 Å) (Fig. 3c). However, isopeptide bonding between Lys47 and Asn172 was no longer visible in the structure. This observation was rather unexpected, as neither of the two corresponding residues was mutated in the N-domain. Upon further inspection of the structure, residues (170–180) encompassing the domain linker region exhibit a 34 Å movement towards the AB loop. This structural rearrangement has caused Asn172 to shift 3.5 Å away from its normal WT position, which subsequently prevents isopeptide bond formation. Moreover, the NH group of the Lys47 side chain shows a ∼160° rotation toward Glu139 and forms a salt bridge. A water molecule lying adjacent to Lys47 is hydrogen-bonded with the Asn172 side chain and the Pro170 main chain. While the Ser149 side chain is rotated towards Lys47, there are some weak electron densities scattered on either side of the Lys47 side chain near the water molecule. These may reflect additional rotamer conformations for this lysine residue. The localized perturbations caused by the displacement of the linker region has disrupted its hydrogen-bonding interactions with the neighboring AB and EF loops, thereby moving each of them away (5 and 7 Å, respectively) from the linker residues. The extended linker region is stabilized by a groove formed by the AB loop with β-strands F and G in the neighboring molecule.

### Structural analysis of single residue-substituted GG-SpaA mutants

Previous studies have suggested that the conserved glutamate of the so-called E-box motif (YxLxETxAPxGY) has a role in minor pilin incorporation, pilus assembly, and internal isopeptide bond formation^3^,^5^,^39^. In GG-SpaA, the N- and C-domains both contain an E-box motif (i.e., ^135^YxxxETxxxxGY^146^ and ^265^YxxxETxxxxGY^276^, respectively). As a means to explore possible roles for the E-boxes in GG-SpaA, single-residue mutants E139A and E269A were produced and then used for crystal structure analysis as well as protein stability measurements. Included in these experiments was also a related third mutant, in which the aspartate that forms the isopeptide bond in the C-domain was substituted with an asparagine (i.e., D295N), the residue that is normally found in Gram-positive backbone pilins. In addition, double mutants E139A/E269A and D295N/E269A were included in the experiments.

### (a) E139A mutant exhibits no isopeptide bond in the N-domain

**Figure 4:**
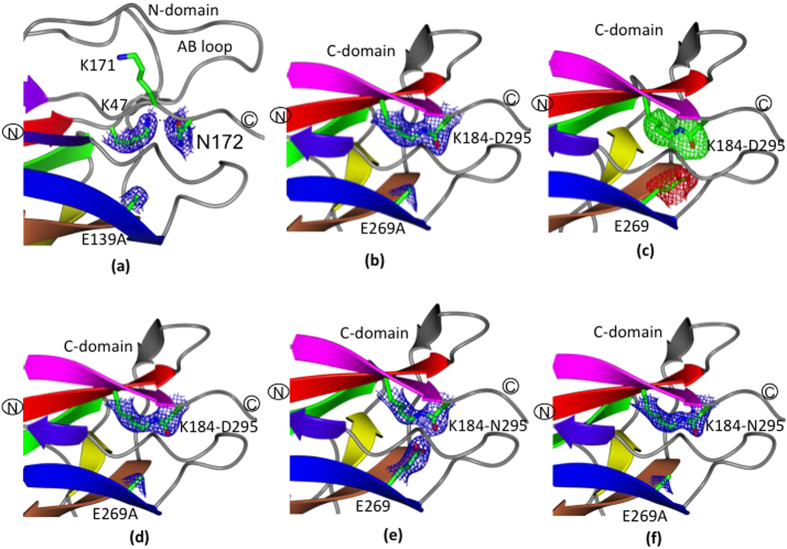
Isopeptide bonds in residue-substituted GG-SpaA. (**a**) E139A mutant. Shown is the absence of the isopeptide bond between K47 and N172 when an alanine is at position 139 in the N-domain. The electron density (2Fo-Fc) map is contoured at 1.5σ. (**b**) E269A mutant. An isopeptide bond is formed between K184 and D295 when an alanine is at position 269 in the C-domain. The electron density (2Fo-Fc) map is contoured at 1.5σ. (**c**) E269A mutant. Difference electron density map contoured at 3σ for the isopeptide bond in the E269A mutant. K184 and D295 were mutated to Ala, and E269 was kept intact in the electron density (Fo-Fc) map calculation. The positive and negative densities are shown in green and red, respectively. (**d**) E269A mutant. An omit map calculated using Sfcheck in the CCP4 suite is contoured at 1.5σ and shows continuous electron density for isopeptide bond between K184 and D295 when an alanine is at position 269 in the C-domain. (**e**) D295N mutant. Shown is isopeptide bond formation between K184 and N295 with glutamate at position 269 in the C-domain. The electron density (2Fo-Fc) map is contoured at 1.5σ. (**f**) D295N/E269A mutant. An omit map calculated using Sfcheck in the CCP4 suite is contoured at 1.5σ and shows continuous electron density for isopeptide bond between K184 and N295 when an alanine is at position 269 in the C-domain.

The overall structure of the E139A-substituted mutant was quite similar to that of the WT GG-SpaA protein (RMSD of 2 Å). Discontinuous electron density was observed between Lys47 and Asn172 that can be attributed to the loss of the N-terminal isopeptide bond (Fig. 4a). The alanine substitution at residue position 139 was also apparent in the electron density map. Despite the overall fold of the E139A mutant being retained, some minor conformational variations are present near the mutation site, including the flexible AB loop region at the N-domain. For instance, individual side chains for the Asn172, Lys47, and Ser149 residues were now oriented differently due to the absence of the isopeptide bond and the subsequently altered hydrogen-bonding network (Fig. 4a). Here, the Asn172 side chain is repositioned closer toward the C-domain and thus able to form a hydrogen bond with the OH group of the Lys47 side chain. Moreover, the Ser149 side chain is rotated towards the domain interface. A water molecule, which is found in the place of the Glu139 side chain, is hydrogen bonded to the NH group of the Asn172 side chain. In addition, the slight shifting of the AB loop backbone has occurred, but with a major structural deviation (3 Å) at Gly69. Because the K-D isopeptide bond was held intact, the structure of the C-domain was seen to be largely unaffected.

### (b) E269A mutant harbors an isopeptide bond in the C-domain

Although the E139A mutant had been isolated using the same purification protocol used for WT protein, these conditions resulted in protein degradation when E269A-substituted GG-SpaA was purified. This was remedied by modifying the purification buffer (see Methods). Here, this allowed for the recovery of intact and pure E269A mutant protein in both a monomeric (E2) and trimeric (E1) form, as evidenced by size-exclusion chromatographic analysis (Supplementary Fig. S1). Crystals of full-length E269A-substituted GG-SpaA had formed after three months for the E1 and E2 protein samples (for details, see Methods and Supplementary Fig. S1), each having the same space groups and similar unit-cell edges as determined for the WT form. Moreover, the overall topological similarity of the E269A crystal structure to that of WT GG-SpaA, and including the molecular arrangement in the asymmetric unit, is high (RMSD of 0.4 Å). Surprisingly, electron density for an intact isopeptide bond is found between Lys184 and Asp295, despite the absence of the glutamate residue at position 269 (Fig. 4b–d).

### (c) D295N mutant contains an isopeptide bond in the C-domain irrespective of E269

D295N-substituted GG-SpaA crystallizes in the same space group as the full-length WT protein and the E139A and E269A mutants, though with different cell dimensions (Table 2). Six molecules in the asymmetric unit were visible, where the N-domain of one molecule is facing the C-domain of an adjacent molecule as in full-length WT. The overall topology of the D295N crystal structure is similar to that of WT GG-SpaA (RMSD of 0.8 Å), with maximum structural deviations found at the AB loop. Significantly, the electron density between Lys184 and the substituted Asn295 clearly supports the formation of an isopeptide bond (Fig. 4e). In D295N, the E269 was also substituted to alanine (i.e., double mutant D295N/E269A) to see its effect on isopeptide bond formation. Interestingly, this additional mutation did not affect the isopeptide bond formation in the C-domain (Fig. 4f).

### Effect of E-box motif mutations on GG-SpaA stability and SpaCBA-pilus polymerization

Numerous studies have reported that the isopeptide bonds in pilin subunits can confer enhanced resilience to various stresses that Gram-positive pili encounter in the environment^7^,^40^. Here, the presence of internal isopeptide bonds within pilins imparts an increased structural stability and rigidity that provides resistance to proteases and higher temperatures^7^,^40^. To assess whether GG-SpaA has these intrinsic properties, proteolytic and thermal stability measurements were performed on the WT form as well as the E139A-, E269A-, and D295N-substituted mutants. Moreover, the effect that GG-SpaA pilin subunits with a residue-substituted E-box have on the assembly of SpaCBA pili was also evaluated.

### (a) Proteolytic stability

**Figure 5:**
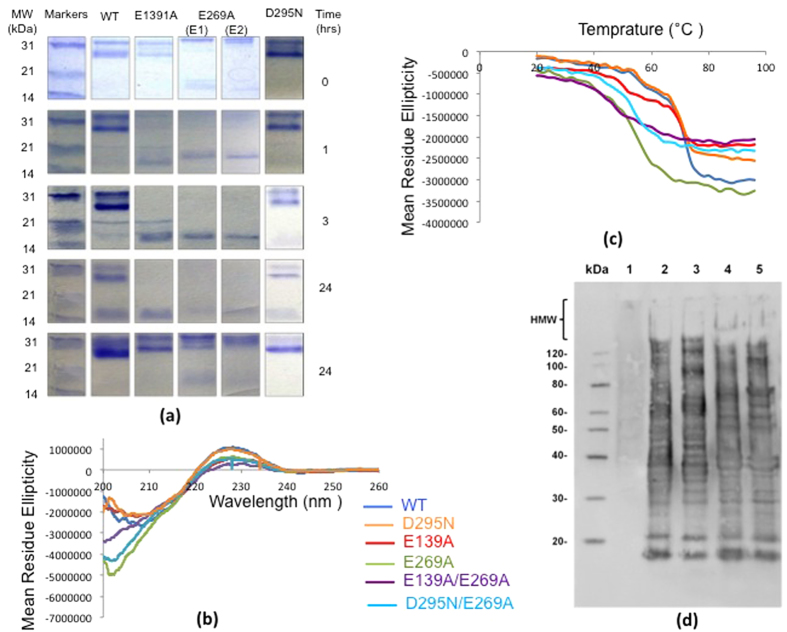
Influence of a residue-substituted E-box on GG-SpaA stability and SpaCBA-pilus polymerization. (**a**) Proteolytic stability of WT and mutant GG-SpaA. Trypsinized GG-SpaA proteins (WT and the E139A, D295N, E269A (E1) and E269A (E2)) were sampled at various time points (0, 1, 3, and 24 h) and analyzed by SDS-PAGE. Molecular weight markers (kDa) are indicated on the left. For clarity, the lanes were cropped from different gels (Supplementary Fig. S2), which have been run under the same experimental conditions. (**b**) Circular dichroism (CD) spectra of WT and mutant GG-SpaA measured at 20 °C over the wavelength range of 200–260 nm. The spectra are colored as follows, WT (blue), D295N (orange), E139A (red), E269A (green), E139A/E269A (violet), and D295N/E269A (cyan). (**c**) Thermal denaturation curves for WT and mutant GG-SpaA. Calculation of thermal stability was based on the change in molar ellipticity at 220 nm as a function of the increased temperature. The 220 nm wavelength near maxima was chosen for making the calculations since there is significant signal noise around the minimum at 207 nm. Curves are colored as in Fig. 5b. (**d**) Recombinant lactococcal cells were nisin induced and analyzed for SpaCBA-pilus production by immunoblotting with anti-GG-SpaA serum. Included are the empty vector GRS1052 (lane 1), WT SpaCBA-piliated GRS1185 (lane 2), E139A-substituted GRS1213 (lane 3), E269A-substituted GRS1215 (lane 4), and E139A/E269A-substituted GRS1217 (lane 5) clones. Compressed high-molecular-weight (HMW) protein bands representing the lengthier pilus fibers along with the molecular weight markers (kDa) are identified to the left of the immunoblot.

As a measure of proteolytic resistance, each type of GG-SpaA protein (WT and mutant) was subjected to trypsin digestion experiments. For this, proteins were incubated with trypsin at 37 °C, with the extent of the treatment assessed over a 24-hour period by SDS-PAGE analysis. Gel lanes were cropped from different gels (Supplementary Fig. S2) and shown side-by-side in Fig. 5a. Here, a protein band (~30 kDa) representing WT and D295N-mutated GG-SpaA was seen to be present throughout the 24 hours of trypsinization, although trace amounts of a smaller ~16 kDa-sized band began to be detected following 3 hours of incubation, with the levels then continuing to increase over time. On the other hand, the E139A and E269A mutant proteins started to quickly degrade, at which point a ~16-kDa band becomes prominently visible within one hour. Moreover, following the 24-hour trypsin treatment, this smaller-sized protein band had nearly disappeared for the E269A mutant, whereas it continued to remain present for the E139A mutant. The results suggest that the resistance to proteolysis apparently displayed by the WT and D295N can be correlated more with isopeptide bond in C-domain than that in the N-domain of GG-SpaA. As observed previously^19^,^28^ the WT protein shows double bands when analyzed by SDS-PAGE. Though the precise reason is not known yet, this aberrant electrophoretic mobility can be attributed to an intramolecular isopeptide bond. Here, the occurrence of a single band for K184A mutant protein (Supplementary Fig. S2g) suggests that this behavior is likely coming from the isopeptide band in the C-domain. Nevertheless, the mutant proteins E139A and D295N, which do not affect isopeptide bond formation in the C-domain, also show a doublet band pattern similar to the WT protein.

### (b) Thermal stability

To estimate the thermal stability of the WT and various residue-substituted GG-SpaA proteins, an analysis of the temperature-dependent circular dichroism (CD) spectra was carried out at pH 7.5. As shown in Fig. 5b, the unheated room temperature CD spectra in the far-UV region for the WT and mutant proteins exhibit a maximum peak at 229 nm, but with minimum peaks observed at 207 nm for the WT form and the E139A and D295N mutants and at 202 nm for the monomeric forms (E2) of E269A and E269A/D295N mutants. The observed peaks in the CD spectra for GG-SpaA and its mutant proteins are different from that of typical β-sheet proteins. Though the exact reason is yet to be recognized, a similar observation has been reported for Spy0128 pilin^41^. Given that the WT, E139A, and D295N proteins share similar spectral profiles, it is quite reasonable to assume that the corresponding isopeptide bonds do not exert a strong influence on the overall secondary structure fold of GG-SpaA. On the other hand, the E269A-substituted and E269A containing double mutants (to a lesser extent) displayed a minimum peak at 202 nm, suggesting the glutamate residue in the E-box of the C-domain might have some role in maintaining proper folding of GG-SpaA. Interestingly, E139A/E269A double-mutant protein, which presumably also lacks the K-N isopeptide bond in the N-domain, displays the smallest maximum peak and has no observable minimum peak.

For assessing the temperature dependence, CD spectra measurements were taken between 20 and 95 °C at 220 nm (Fig. 5c). This particular wavelength was chosen for the ease of visualizing the transitions, as the spectra around the minima are noisy. CD spectra were also recorded from 200 to 260 nm for the GG-SpaA proteins (WT and mutants) when in both the folded (before heating to 95 °C) and refolded states (after cooling down to 20 °C) (Supplementary Fig. S3). For the WT and mutant proteins, the CD signal exhibited a gradual reduction as the temperature is increased. For WT, E139A, and D295N GG-SpaA, there appears to be a transitional temperature (*T*_*m*_) that is not apparent for the E269A-, D295N/E269A-, and E139A/E269A-substituted proteins. Here, a first and second *T*_*m*_ for both the WT and D295N proteins was observed at around 52 and 66 °C, respectively. An approximately 5 °C decrease in *T*_*m*_ was seen in the first transition for the E139A-substituted protein. The monomeric E269A (E2) sample displayed only one transition point, with this being detected at around 38 °C, while the trimeric E269A (E1) sample is seen to be similar but with some additional signal noise. Concerning the temperature-dependent CD spectral changes for the double-mutant proteins, D295N/E269A appeared comparable to that of E269A, whereas for E139A/E269A this substituted protein was lacking any obvious transition points with a rapid reduction. For nearly all GG-SpaA proteins being examined, there are no significant differences in the far-UV CD spectra between the native state and the cooled-down heat-denatured samples (Supplementary Fig. S3). There was also no precipitation of the protein at the higher temperatures. This suggests that the various residue-substituted mutations cause no adverse effects during thermal-induced protein unfolding/refolding processes. In this regard, only E139A-substituted protein at the minimum peak had offered any significant aberration between the two measured states.

### (c) SpaCBA-pilus polymerization

To study the effect of the mutations on pilus polymerization, a recombinant WT SpaCBA-piliated *Lactococcus lactis* construct^25^ was mutated to contain backbone pilins having the E139A, E269A, and E139A/E269A residue substitutions (GRS1213, GRS1215, and GRS1217, respectively). The impact on polymerization in the corresponding lactococcal clones was then determined by immunoblotting analysis using anti-GG-SpaA serum. Here, production of the various-sized pili is detected as a laddered length of protein bands, including those that are higher molecular weight and compressed. As shown in Fig. 5d, the immunoblotting pattern for the E-box mutated SpaCBA piliated lactococci (lanes 3–5) is the same as for the recombinant clone expressing WT pili (GRS1185) (lane 2). This result suggests that while the E139A, E269A, or E139A/E269A residue substitutions can structurally affect the individual GG-SpaA subunit (see preceding sections), the removal of the E-box glutamate residue does not exert a cumulative effect to disrupt the normal backbone-polymerization of SpaCBA pili.

### Comparison of GG-SpaA with other pilin structures

**Figure 6:**
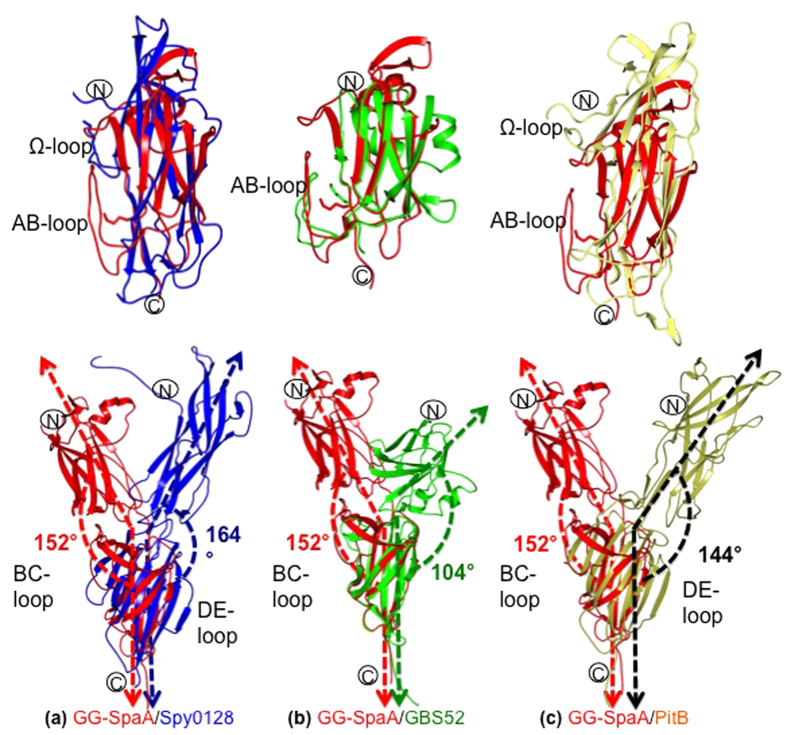
Structural comparison of GG-SpaA with other two-domain pilins. Superposition of GG-SpaA (red) on Spy0128 (blue) (**a**), GBS52 (green) (**b**), and PitB (gold) (**c**). Upper structures are of superimposed N-domains. In the superposition of GG-SpaA and GBS52, the linking lysine (in sticks) and the AB loop are positioned similarly. The linking lysine and the Ω loop of Spy0128 and PitB are positioned midway along their structures. Lower structures are of full-length protein, with the C-domains superimposed and the N-domains displaced. Major structural perturbations are at the BC and DE loops in the C-domains.

Through the DALI server^42^, a search for structural homologs of GG-SpaA in the PDB had identified many matching pilin-proteins and surface adhesins from various Gram-positive pathogens (Supplementary Table S1). Although Spy0128 from *S. pyogenes* is a two-domain pilin with CnaB folds^5^, it was not among the hits identified in the DALI PDB search. For Spy0128, because of existing twists and bends between the two domains, any alignments of the core β-strands of the CnaB fold in one domain that are done on the basis of secondary structural elements prevent a proper fit with the other domain (Fig. 6a). However, the core β-strands of the CnaB fold exhibits a much better alignment if the superposition of the N- and C-domains is done separately. This also appears to be true for GBS52^4^, a two-domain basal pilin consisting of CnaB folds (Fig. 6b).

Secondary structure matching (SSM) superposition of the GG-SpaA N-domain on Spy0128 and GBS52 was done using *COOT* 43 (Fig. 6a,b, respectively). Any structural deviations are confined to the loop regions, primarily with the AB and BC loops. The AB loop extending over the lysine residue that links together the pilin subunits is seen to be similar in GG-SpaA and GBS52. However, in the case of Spy0128, an extended AB loop is missing and in its place there is an omega (Ω) loop in the middle of last β-strand of the N-domain that contains the protrusive pilin-linking lysine. Here though, the β-sandwich core in the GG-SpaA structure (including that of GBS52) does not appear as elongated as in Spy0128. When the GG-SpaA C-domain is SSM superimposed with Spy0128 and GBS52 (Fig. 6a,b, respectively), most of the structural aberrations are associated with the BC loops. For instance, the two β-strands of the BC loop are aligned perpendicular to the core β-strands in GG-SpaA, as opposed to running in parallel as in Spy0128. As well for Spy0128, the extending EF loop causes two β-strands to align with core β-strands on the opposite side of the BC loop. However, in GG-SpaA (and GBS52) the EF loop has a shorter length and cannot exert the same effect. Moreover, the structural superposition of the GG-SpaA C-domain on Spy0128 and GBS52 causes a rotational displacement of ~70° at the N-domains (Fig. 6a,b, respectively), and where the bendable inter-domain hinge angle^31^ for GG-SpaA, Spy0128, and GBS52 is 152°, 164°, and 103°, respectively. Of particular note, while writing this manuscript the structure of the *S. pneumoniae* PitB backbone pilin was published^44^. PitB also has a two-domain arrangement with an elongated CnaB fold as seen in Spy0128, and when superimposed with GG-SpaA closely resembles that with Spy0128 (Fig. 6c).

### Structural model for SpaCBA-pilus assembly

**Figure 7:**
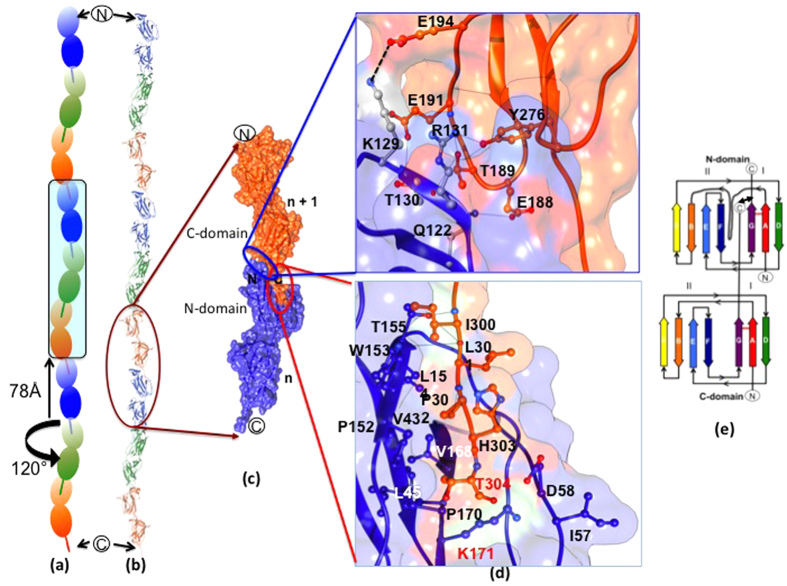
Structural model of assembled GG-SpaA pilin subunits. (**a**) Schematic representation of the GG-SpaA head-to-tail arrangement in the SpaCBA pilus. Shown is the pilus-like assembly of repeating pilin molecules (orange, green, and blue) as based on the molecular packing of the GG-SpaA crystal (see Supplementary Fig. S4). Three pilin molecules in the asymmetric unit are highlighted. Indicated is the 120° rotation and 78 Å translation with each successive GG-SpaA pilin molecule along the pilus axis. (**b**) Cartoon representation of GG-SpaA pilin subunits in an assembled SpaCBA pilus structure, as deduced from the molecular packing of crystals (see Supplementary Fig. S4). Coloring of pilin subunits is as given above. (**c**) Molecular surface representation of two adjacent GG-SpaA pilin subunits in the assembled SpaCBA pilus structure. The LPXTG sorting motif of the C-terminal tail from one subunit being docked into the pilin motif-containing groove of another subunit is shown. (**d**) An enlarged view of the molecular interactions between two adjoining GG-SpaA pilin subunits. Upper panel shows the inter-domain interface between the C-domain one subunit (*n* + *1*) and N-domain of the neighboring subunit (*n*). Lower panel shows the molecular interface between the threonine of the C-terminal LPXTG sorting motif of the *n* + *1* subunit and the essential lysine of the pilin motif-containing groove formed by the AB loop in the N-domain of the *n* subunit. Modeling of H303 and T304, which are not part of the crystal structure, brings the K171 within a distance sufficient for a covalent bond. Interface residues are depicted in ball-and-stick representation. Hydrogen bonds and salt bridges are shown as solid and dashed black lines, respectively. (**e**) Topology diagram showing the insertion of the C-terminal tail of one pilin into the N-domain of the adjacent pilin. The β-strands A- G are rainbow colored as in Fig. 1c. A double-headed arrow indicates the location of an intermolecular covalent cross-link.

Intriguingly, the three molecules present in the asymmetric unit of the GG-SpaA crystal are seen to generate what resembles a pilus-like fiber (Supplementary Fig. S4), and where the corresponding pilin molecules are in a “head-to-tail” arrangement that may reflect its native biological state (Fig. 7). As they are arranged, this places the C-terminal sorting motif of each molecule into a cavity-like space nearby the pilin motif of the adjacent molecule. These interactions then conceal a surface area of ~1300 Å^2^ that lies between the C- and N-termini of neighboring GG-SpaA molecules (Fig. 7c,d). Along the axis of the pilus structure, each of the GG-SpaA molecules shows a rotation angle of ~120° and translation of ~78 Å. The inter-domain hinge angle (152°) in each GG-SpaA molecule and the head-to-tail positioning of adjoining molecules allow for the formation of an elongated spiral staircase-like arrangement that could then offer both flexibility and rigidity to SpaCBA pilus structure. With this particular arrangement, the solvent content of the crystal is 70%, and a large solvent channel is clearly seen running down the z-axis (Supplementary Fig. S4). Up to now, this high solvent content has not been described for crystals of Gram-positive pilin-proteins, although some of them exhibit a similar pilus-like fiber in the crystal packing^5^,^29^,^44^,^45^.

## Discussion

Our findings show that the GG-SpaA structure has certain features in common with other pathogen-derived pilin-proteins, such as the Ig-like CnaB-type folds and the presence of internal isopeptide bonds^6^,^9^,^40^. For instance, the domain organization of GG-SpaA bears a structural resemblance to the two-domain backbone-pilin subunits Spy0128 from *S. pyogenes*^5^ and PitB from *S. pneumonia*e^44^, but with the hinge angles noticeably different. Of the two domains in GG-SpaA, the N-domain contains an isopeptide bond that forms between lysine and asparagine residues (K-N), a type commonly seen in several other Gram-positive pilins. This contrasts with the C-domain of GG-SpaA, where an isopeptide bond is also present, but is instead between lysine and aspartate residues (K-D). Thus far, this form of bond pairing has not been seen in any other backbone pilin structures. While the two types of isopeptide bonds are formed by a similar mechanism, with some environmental factor helping to shape the evolutionary selection of either an aspartate or asparagine residue^35^,^36^, the reason for the slower forming K-D isopeptide bond in GG-SpaA is not so obvious, but might possibly stem from an adaptive need that the gut milieu imposes on SpaCBA-piliated *L. rhamnosus* GG cells. With interest, based on the structural comparisons of the GG-SpaA pilin with other pilins from pathogen hosts, it appears that there are no topologies in the tertiary structure of these proteins that would distinguish a “virulence factor” pilus from a “niche-adaptation factor” pilus. Thus, in the context of any structural role, pathogen- and non-pathogen-derived backbone-pilin subunits seem more similar than different. Nevertheless, as GG-SpaA forms the polymerized backbone, the main distinguishing features of the SpaCBA pilus might lie with the ancillary pilins^27^, but whose structures are yet to be determined.

Intriguingly, the alanine substitution of the C-terminal E-box glutamate (E269A) that presumably catalyzes K-D isopeptide bond formation had shown two contrasting structural effects. Here, this E-box mutation initially produced a crystal structure of the truncated N-domain lacking the K-N isopeptide bond (see further below), but then afterward, with the overall fold being retained where an intact K-D isopeptide bond was still visible in the C-domain. Up till now, this latter bonding behavior had never been observed, as in all known pilin structures, either a glutamate or an aspartate found in the vicinity of an isopeptide bond is directly associated with the bond-making process. However, whether this is unique to the K-D isopeptide bond in GG-SpaA, or then a universal attribute of other Gram-positive pilins and surface proteins remains uncertain and will require further study.

Because the N-terminal domain of the various Gram-positive backbone pilins is flexible in nature (for a recent list, see ref. 6), it tends to hinder the crystallization process. Consequently, most pilin-proteins can only crystallize when the N-terminal domain has been removed, either spontaneously or through limited proteolysis. As an outcome of this, no crystal structure of the N-domain alone has been solved for a Gram-positive pilin, although there is one report of an NMR structure^46^. Now for the first time, we were able to produce diffractable crystals that included the N-domain of GG-SpaA, and with them also solve the structure of this domain. From the structure it appears that the C-terminal tail of GG-SpaA (β-strand G) must be properly positioned to create a favorable molecular environment that then facilitates isopeptide bonding in the N-domain. As such, this seems only possible when the C-domain (or its equivalent mimicking region in crystal packing) is present. This explains why the loss of the GG-SpaA C-domain via the E269A substitution affected the N-domain isopeptide bond formation and the local network of bond interactions. Additionally, structural studies involving the RrgB and BcpA pilins suggest that the presence of the LPXTG sorting motif and actively assembling pilin subunits are needed as well^45^,^47^. Moreover, the pilin motif and E-box in the N-terminal domain of most backbone and some basal pilins are in close proximity to each other, suggesting the involvement of the E-box during isopeptide bond formation and pilus polymerization. By comparison, the well-conserved C-terminal domain E-box in the backbone and tip pilins, in addition to having a role in forming isopeptide bonds, is also important for protein folding and conformational stability, which in itself is crucial for the pilus-assembly process.

One conundrum about the *L. rhamnosus* GG SpaCBA pilus that remains unresolved is whether its backbone structure also contains the ancillary pilins, with these being either incorporated within or decorated externally. As interpreted visually from earlier immuno-electron microscopic analyses^11^,^16^ there are indications that the basal GG-SpaB and the tip-localized GG-SpaC pilins are also positioned along the length of the pilus. This contrasts with the findings of a more advanced examination involving cryo-electron microscopy of the *S. pneumoniae* pilus, as here the ancillary pilins are found nowhere else than at the pilus base or tip^48^. However, as the basal pilins can also contain the conserved residues of the pilin motif (YPKN), which means they have the lysine needed for linking to the threonine residue of another pilin-protein, this type of subunit has the possibility of being randomly misincorporated into the elongating pilus, as had been earlier described for pilus assembly in corynebacteria^49^. In our present study, molecular packing in crystals offers evidence that the GG-SpaA pilins could alone form the pilus backbone. There is, though, no structural basis for “atypical” incorporation of ancillary pilins. Nonetheless, with the presence of the YPKN-like pilin motif in the GG-SpaB pilin, this subunit could in fact be positioned in the pilus in a structurally analogous way as seen with the GG-SpaA N-domain or as evidenced by the similar structural analysis of the GBS52 4,50 and RrgC^51^ pilins. Still, while there is some supportive mechanistic justification for GG-SpaB integration within the backbone structure of the SpaCBA pilus, this would not be the case for the adhesive GG-SpaC tip-pilin. Because the canonical pilin motif is not evident in the primary structure of GG-SpaC, this pilin-protein lacks the critical lysine residue deemed necessary for adjoining to any neighboring subunit. Moreover, while the crystal structure of GG-SpaC is yet to be solved, our scrutiny of the GG-SpaA structure did not reveal any obvious structural topologies that might lend support to a molecular mechanism that could then explain the perceived GG-SpaC adornment of the pilus backbone^11^,^16^.

In summary, we have revealed the first crystal structure of a pilin subunit (GG-SpaA) from a non-pathogen host, namely gut-adapted *L. rhamnosus* GG. The domain fragments derived from limited proteolysis had helped us in solving the crystal structure of full-length GG-SpaA. For the most part, the two isopeptide bond-containing CnaB domains of GG-SpaA are similar to their counterparts in pathogenic strains. However, differences with the loop regions, isopeptide bond formation, and domain orientations were identified. The E-box glutamate residues (E139 and E269) seem to have some effect on the proteolytic and thermal stability of GG-SpaA, but do not affect the pilin structure and pilus polymerization. Surprisingly, the mutation of E269 in the C-domain had no influence on K-N or K-D isopeptide formation. Fortuitously, the E269A mutation allowed us to capture the crystal structure of a truncated N-domain, which, in pathogen hosts, is known to be labile and has never been before solved by X-ray crystallography. Conformational changes observed in the N-domain provide new structural insights into how the movement of adjacent domains can affect isopeptide bond formation, and then what possible outcome this might have on pilus stability and polymerization. As well, based on the molecular packing of crystals, we have been able to propose a model that shows how the polymerized GG-SpaA pilins are arranged to form the backbone structure of the SpaCBA pilus. Finally, our ongoing structural investigations of the GG-SpaB and GG-SpaC ancillary pilins should shed further light into the assembly and properties of *L. rhamnosus* GG SpaCBA piliation.

## Methods

### Protein production

Full-length *L. rhamnosus* GG (ATCC 53103) SpaA_35–302_ (GG-SpaA) containing a C-terminal His-tag was produced recombinantly in the *E. coli* BL21 (DE3) pLysS strain^19^, and then purified by nickel-affinity and size exclusion chromatography as described earlier^28^. Briefly, a cell-free lysate was applied onto a HiTrap chelating HP column (GE Healthcare) equilibrated with 40 mM NaH_2_PO_4_ buffer (pH 7.4) containing 400 mM NaCl. Bound GG-SpaA protein was then eluted by a linear gradient of the same buffer that also contained 400 mM imidazole. Size-exclusion chromatography was carried out using a Sephacryl S-200 26/60 column (GE Healthcare) equilibrated with 20 mM HEPES buffer (pH 7.0) containing 150 mM NaCl and 1 mM EDTA. Eluted fractions judged to contain pure protein were pooled and concentrated for crystallization and further analysis. Purification of GG-SpaA mutant proteins was done using a similar strategy, but with some modifications to the buffer composition for the E269A and double mutants. For the E139A/E269A and D295N/E269A double-mutant proteins, 1 M imidazole was required in the elution buffer during nickel-affinity purification as these proteins had bound strongly to the resin. For E269A, E139A/E269A, and D295N/E269A proteins, 20 mM Tris buffer (pH 7.5) supplemented with 150 mM NaCl and 1 mM EDTA was used during size exclusion chromatography.

### Limited proteolysis

Purified GG-SpaA protein was first subjected to limited proteolysis using various proteases and analyzed by SDS-PAGE. However, the use of α-chymotrypsin resulted in a stable 15 kDa-sized fragment when incubated with a protease/protein ratio of 1:100 for 36 hours at 37 °C in a buffer containing 20 mM HEPES (pH 7.5), 150 mM NaCl, 1 mM HCl, and 2 mM CaCl_2_. Chymotrypsin-digested GG-SpaA fragments containing 0.5 mM PMSF protease inhibitor were subjected to size exclusion chromatography using a Sephacryl S200 26/60 column containing a buffer of 20 mM HEPES (pH 7.5) and 150 mM NaCl, and then concentrated to 35 mg/ml for crystallization screening trials.

### Site-directed mutagenesis

Site-directed mutagenesis by PCR was used to introduce nucleotide changes into the *spaA* gene. For this, a separate set of mutagenic forward and reverse primers were designed (see Supplementary Table S2), with the *spaA*-bearing pKTH5319 construct^19^ used as the DNA template. PCR amplification was carried out using Pfu Turbo DNA polymerase (Stratagene). Amplified DNA was treated with DpnI at 37 °C to digest the template strand, which was then transformed into XL10-GOLD ultra-competent cells (for single mutants) or the *E. coli* DH5α strain (for double mutants). Transformants with the desired mutations were confirmed by DNA sequencing.

### Crystallization and data collection

WT GG-SpaA protein had initially crystallized under several conditions^28^. However, crystals from one of the optimized conditions (0.3 M tri-sodium citrate and 15% PEG 3350) containing a 1M lithium acetate as a cryoprotectant showed an improved X-ray diffraction quality. The chymotrypsin-digested 15 kDa GG-SpaA fragment (C-domain) and other mutant proteins were crystallized initially by the sitting drop method at room temperature under different crystal screening conditions. Those conditions that appeared promising were further optimized by using the hanging drop method. Good-quality crystals were obtained after 15 days for the C-domain. Here, drops containing 2 μl protein and 1 μl reservoir solution were equilibrated against 800 μl reservoir solution. The 15 kDa fragment was crystallized in two different conditions. Rock-shaped crystals produced in a solution containing 0.1 M MES (pH 6.5), 0.02 M zinc sulphate, and 25% PEG 550MME belonged to the orthorhombic space group and diffracted to 2 Å at a home source (Regional Centre for Biotechnology, India). Rod-shaped crystals were generated in a solution of 0.1 M sodium acetate (pH 4.6) and 25%PEG 4000 and belonged to the tetragonal space group. This crystal form diffracted to 2 Å at a home source (Regional Centre for Biotechnology, India), and to 1.6 Å at a synchrotron source (BM14 beamline at the ESRF, Grenoble). Ethylene glycol (20% v/v) served as the cryoprotectant for both the orthorhombic and tetragonal crystal forms.

E139A and E269A mutant proteins were concentrated (120 and 96 mg/ml, respectively) and screened for crystal growth. E139A crystals were obtained in a solution of 0.1 M sodium thiocynate and 15% PEG 3350 after one week at 4 °C. Rod-shaped crystals of monomeric (E2) E269A protein were obtained in a solution of 0.2 M sodium formate and 20% PEG 3350 after two weeks at room temperature. These crystals represented the N-domain portion of GG-SpaA, and diffracted to 2 Å (home source: National Institute of Immunology, India). Crystals of the monomeric (E2) and trimeric (E1) E269A mutant proteins were observed in a 0.2 M tri-potassium citrate and 20% PEG 3350 solution after three months at room temperature, with these representing full-length GG-SpaA. Crystals for D295N mutant were obtained in 0.1 M Tris-HCl (pH 8.5) and 15% PEG20000 in a week. D295N/E269A crystals were observed in a solution containing 0.1 M sodium/potassium tartrate, 100 mM Tris (pH 7.0), 200 mM lithium sulphate after two weeks at room temperature. For X-ray diffraction experiments with the E139A, E269A, D295N, and D295N/E269A crystals, 30% ethylene glycol in mother liquor was used as the cryoprotectant. Diffraction data from E139A, E269A (E2), E269A (E1), D295N, and D295N/E269A crystals were collected at synchrotron source (BM14 beamline at the ESRF, Grenoble). X-ray diffraction data from WT and E139A crystals were processed with HKL2000^52^. Processing and scaling of data from other crystals was done by XDS^53^ and Aimless^54^.

### Structure solution and refinement

Crystals of full-length WT GG-SpaA protein belonged to space group *C*2 with the unit cell dimension of a = 228.0, b = 63.2, c = 104.3 Å, and β = 95.1°. Calculation of the Matthews coefficient (VM = 2.44 Å^3^ Da^−1^) for a solvent content of 50% suggested the presence of five molecules in the asymmetric unit. Initial attempts to solve the crystal structure by molecular replacement (MR) and heavy-atom derivative methods were unsuccessful. Alternatively, phase calculations were feasible by SAD and MR methods for the C-domain of GG-SpaA, which had been generated by limited proteolysis, with crystals produced in the orthogonal and tetragonal forms. Orthogonal crystals belonged to space group *P*2_1_2_1_2, having the unit cell dimension of a = 57.1, b = 74.3, c = 116.8 Å and containing four molecules in the asymmetric unit, based on a Matthews coefficient of 2.36 Å^3^ Da^−1^ and an estimated solvent content of 48%. Phase calculations performed in *PHENIX.AUTOSOL*^55^ using SAD data collected from an ytterbium (Yb)-derivatized crystal had allowed structure solution from ten identified heavy atom positions (figure of merit = 0.32) and built 80% of the model (R_work_/R_free,_ 30.4%/38.7%). Complete model building and refinement of the 15 kDa-sized GG-SpaA fragment containing residues 176–298 and corresponding to the C-domain had resulted in a R_work_/R_free_ of 20.8%/25.3%. Inclusion of water molecules and metal-ions in the final cycles of refinement (*REFMAC*^56^) had reduced the R_work_/R_free_ to 19.7%/23.6%. This structural model was then used in MR for the native data.

A parallel attempt to obtain structure solution by the MR method had yielded an interpretable electron density for the tetragonal crystals of the C-domain. These crystals belonged to space group *I*422 with the unit cell dimension of a = 100.2, b = 100.2, c = 57.5 Å and one molecule in the asymmetric unit, as per the Matthews coefficient calculation (VM = 2.76 Å^3^ Da^−1^) and a solvent content of 55%. Structure solution was obtained by the *PHENIX.MR_ROSETTA* algorithm tool in *PHENIX*^55^. It generated multiple search templates using sequence information from the Protein Data Bank (PDB) (i.e., 3PF2, 2X5P, 4HSQ, 2XI9, 3PHS, 2XTL, and 2WW8), which was then used in the MR calculations. The best solution was with the search model from 3PHS, which represents the *S. agalactiae* GBS52 ancillary pilin^4^. This solution was used as a starting point, with further trimming of the model based on the density, which then yielded a R_work_/R_free_ of 46.3%/49.9%. Several cycles of refinement using *REFMAC*^56^ and model building using *COOT*^43^ from the *CCP4* package^57^, along with the inclusion of water molecules, had given a R_work_/R_free_ of 13.9%/15.8%. Residues for the C-terminal hexahistidine tag along with six residues preceding it were not observed in the electron density map, and either disordered or lost during the limited proteolysis of GG-SpaA.

The C-domain model was used to solve the full-length structure of GG-SpaA. Initially, three copies of the C-domain were placed, and then multiple templates from the PDB (i.e., 2L4O, 3UXF, 3HR6, 2Y1V, and 4HSS) generated for the N-domain using the corresponding sequence information were then used to place three copies of N-domain for automated model building with *BUCCANEER*^58^ in the CCP4 package. Placement of the search model from 2L4O (D1 domain of the *S. pneumoniae* RrgB pilin^46^) had yielded a R_work_/R_free_ of 30.2%/33.8%, which was lowered further to 22.8%/25.3% in the final cycle of refinement following the inclusion of water molecules. As mild anisotropy in the diffraction pattern was noticed, a correction was made by using an anisotropic server (http://services.mbi.ucla.edu/anisoscale/)^59^. By using the anisotropically truncated diffraction data in the refinement (*PHENIX*), this had lowered the R_work_/R_free_ to 17.4%/21.7%. The final model of full-length GG-SpaA consisted of residues 39–302. Four N-terminal residues (35–38) and the residues of the C-terminal His-tag (LEHHHHHH) are encoded by the expression plasmid and were not modeled into the structure due to insufficient electron density. Similarly, residues 50–60 (AB loop) could not be modeled in molecules B and C due to a lack of electron density, although they could in molecule A where there was clear electron density.

The crystal of E139A mutant protein belonged to the *C2* space group with the unit cell dimension of a = 223.9, b = 63.5, c = 104.5 Å, and β = 94.9° and then rather similar to that of the full-length WT GG-SpaA crystal, having three molecules in the asymmetric unit with a Matthews coefficient of 4.27 Å^3^ Da^−1^ and a solvent content of 71.2%. The structure was solved by the MR method using WT GG-SpaA as the search model. Iterative cycles of refinement were carried out with *REFMAC*. The final refined model of E139A-substituted GG-SpaA consists of residues 38 to 302 and had yielded a R_work_/R_free_ of 20.1%/21.7%.

The crystal of E269A mutant (N-domain) protein belonged to the *P* 4_1_2_1_2 space group with the unit cell dimension of a, b = 88.6 and c = 48.8 Å, which differs from that of the WT and E139A-substituted GG-SpaA crystals. The calculated Matthews coefficient (VM = 1.66 Å^3^ Da^−1^) for the full-length protein (35–302) suggested the presence of a single molecule in asymmetric unit with a 26% solvent content, which is considered highly unusual. Initial attempts to solve the structure by the MR method using WT GG-SpaA as the search model did not succeed. As a remedy to this, search models with the N- and C-domains were done separately in the MR calculations. Here, a solution was obtained with the N-domain as the search template. Refinement was carried out with *PHENIX*^60^ at 1.9 Å resolution, and the final model consisting of residues 38 to 180 and having yielded a R_work_/R_free_ of 18.5%/22.2%. Residues 181 to 302 that correspond to the C-domain had lacked electron density and could not be modeled into the structure. This region might have been truncated away as result of the absent stabilizing C-terminal isopeptide bond and/or from *in-situ* protein degradation during the crystallization process.

Analogous to WT and E139A-substituted GG-SpaA, the crystals of full-length monomeric (E2) and trimeric (E1) E269A protein both belonged to the *C2* space group with unit cell dimension of a = 228.6, b = 63.0, c = 104.9 Å, and β = 95.3° and have three molecules in the asymmetric unit. The same strategy as with E139A crystals was used in the MR and refinement of monomeric E269A, which yielded a R_work_/R_free_ of 21.9%/23.7% for the final model consisting of residues 38–302. The R_work_/R_free_ of 25.2%/26.9% was obtained for the trimeric E269A model (further data is not shown since the model is identical to that obtained for the monomeric E269A protein).

D295N crystals belonged to the *C2* space group with unit cell dimension of a = 164.3, b = 75.2, c = 176.5 Å, and β = 100.3°, but having six molecules in the asymmetric unit. Pseudo translation symmetry was detected, with the structure being solved by molecular replacement using *PHASER-MR* in *PHENIX*. After including water molecules, the final model (residues 38–302) yielded a R_work_/R_free_ of 24.2%/26.5%. Electron density is weak for one of the molecules in the asymmetric unit. D295N/E269A crystals belonged to *C*2 space group and cell-dimensions are similar to that of full-length WT crystals.

### Proteolytic assay

WT and mutant GG-SpaA proteins were dissolved in 25 mM ammonium bicarbonate to a final concentration of 1 mg/ml and then treated with trypsin at a ratio of 1:100 (trypsin:protein, w/w) at 37 °C. Aliquots (20 μl) were taken at different time intervals (0, 1, 3, and 24 h) and supplemented with 10 μg (per sample) trypsin/chymotrypsin inhibitor from Glycine max (Sigma). The extent of proteolysis on the GG-SpaA proteins was analyzed electrophoretically on 15% SDS-polyacrylamide gels. The untreated GG-SpaA proteins incubated in the same temperature (37 °C) were also taken at different time intervals for control. The gels (Supplementary Fig. S2) were run using the same experimental conditions and the corresponding lanes were cropped for clarity (Fig. 5a).

### Circular Dichroism (CD) Spectroscopy

WT and mutant GG-SpaA proteins were subjected to CD analysis on a JASCO J-815 CD spectrophotometer fitted with a Peltier based temperature control unit. Proteins were dissolved in 25 mM NaH_2_PO_4_ (pH 7.5) buffer to a final concentration of 0.4 mg/ml, with a 300 μl volume aliquot added to a quartz cuvette (1-cm path length). CD spectra were recorded from 200 to 260 nm at 20 °C using a 1.0 nm bandwidth and a data interval of 0.1 nm with a signal averaging time of 1 sec. For thermal melting experiments, CD spectra were measured at 220 nm with a gradual increase from 20 to 95 °C at 0.5 °C intervals with a scan rate of 1 °C/min. Protein refolding after thermal denaturation was assessed by heating samples to 95 °C and then cooling them down to room temperature. Raw data units were converted to mean residue ellipticity after the background absorbance signal was corrected. To calculate the thermal denaturation profile, changes in mean residue ellipticity were plotted as a function of temperature. The transitional temperature (*T*_*m*_) of proteins was determined from the inflection point of the melting curve.

### Immunoblotting analysis of E-box mutated SpaCBA-piliated lactococci

The recombinant GRS1185 lactococcal clone that produces nisin-inducible WT *L. rhamnosus* GG-SpaCBA pili was constructed previously^25^ and used as the positive control during immunoblotting experiments. Its fimbrial *spaCBA* operon-bearing expression plasmid (pKTH5391) served as the DNA template for generating the mutant clones that contain E139A-, E269A-, or E139A/E269A-substituted GG-SpaA pilin-protein. The necessary nucleotide changes (GAA→GCA) were introduced into the *spaA* locus by using overlap extension PCR and then verified afterward by DNA sequencing. Mutagenized plasmids (pKTH5414 for E139A, pKTH5417 for E269A, and pKTH5418 for E139A/E269A) were reintroduced into the *Lactococcus lactis* NZ9000 strain much in the same way as was described previously^25^. The E-box mutated SpaCBA-piliated lactococcal clones were named GRS1213 (E139A), GRS1215 (E269A), and GRS1217 (E139A/E269A). The GRS1052 lactococcal clone carrying a plasmid without the *spaCBA* operon insert that had been made previously (pKTH5080; unpublished) was used as a negative control. Overnight nisin-induced expression of the recombinant lactococcal clones (GRS1052, GRS1185, GRS1213, GRS1215, and GRS1217) was performed as described elsewhere^13^,^17^,^25^. Immunoblotting was done using conventional procedures. For this, lactococcal cells were pelleted centrifugally, washed once with phosphate-buffered saline, and afterward resuspended in the same buffer. Each cell suspension was diluted 1:1 with Laemmli buffer, heated to 100 °C for five minutes, and then clarified by centrifugation. SDS-electrophoresis on a precast 4–20% gradient gel (Sigma-Aldrich) was used to resolve the protein content in the samples, which was then electroblotted onto a nitrocellulose membrane (0.45 μm pore size; Bio-Rad). The membrane was treated with rabbit anti-GG-SpaA serum (diluted 1:5000)^19^, followed by horseradish peroxidase-conjugated goat anti-rabbit IgG (diluted 1:10,000; Bio-Rad). Protein bands were visualized using a chemiluminescent detection kit (Western Lightning Plus-ECL; Perkin Elmer, Inc) as specified by the manufacturer.

## Additional Information

**Accession codes:** The atomic coordinates of the models and their corresponding structure factors have been deposited in the Protein Data Bank (www.pdb.org) with the entry codes 5F44 (WT full-length), 5FAA (WT C-domain, tetragonal form), 5FGS-(WT C-domain, orthorhombic form with Zn), 5FGR (WT C-domain, orthorhombic form, Yb derivative), 5FIE (E269A (N-domain)), 5HBB (E139A), 5HDL (E269A-E2), 5HTS (D295N), and 5J4M (D295N/E269A).

**How to cite this article**: Chaurasia, P. *et al*. New insights about pilus formation in gut-adapted *Lactobacillus rhamnosus* GG from the crystal structure of the SpaA backbone-pilin subunit. *Sci. Rep.* **6**, 28664; doi: 10.1038/srep28664 (2016).

## Supplementary Material

Supplementary Information

## Figures and Tables

**Table 1:** Crystallographic data of full-length GG-SpaA and its C- and N-domain*.

	Full-length	C-domain (tetragonal)	C-domain (orthorhombic-native)	C-domain (orthorhombic–Yb derivative)	N-domain (E269A)
Beamline	BM14-ESRF	BM14-ESRF	Cu Kα radiation	BM14-ESRF	Cu Kα radiation
Wavelength (Å)	0.97625	0.97625	1.54178	1.28136	1.54178
Resolution range (Å)	32–1.90 (1.97–1.9)	50.0–1.6 (1.66–1.6)	50–2.0 (2.07–2.0)	50–2.8 (2.89–2.79)	44.31–2.0 (2.07–2.0)
Space group	*C* 2	*I* 4 2 2	*P* 2_1_ 2_1_ 2	*P* 2_1_ 2_1_ 2	*P* 4_1_ 2_1_ 2
Unit cell constants a, b, c (Å); β (°)	227.97, 63.2, 104.27; 95.14	100.24, 100.24, 57.54	57.07, 74.34, 116.78	57.60, 75.25, 117.39	88.61, 88.61, 48.89
No. of Unique reflections	84072 (7273)#	19599 (1916)	33951 (3222)	13174 (1254)	13618 (1330)
Multiplicity	6.5 (4.1)	28.5 (29.1)	9.2 (8.8)	14.0 (13.8)	6.9 (6.9)
Completeness (%)	72.49 (10.58)#	99.80 (99.64)	99.19 (96.18)	99.77 (97.82)	99.67 (99.92)
Mean I/σ (I)	17.91 (3.14)	36.96 (4.83)	24.26 (2.54)	22.75 (6.52)	11.76 (3.27)
Wilson B-factor (Å^2^)	15.93	17.21	32.11	53.77	19.46
R-merge	0.08 (0.44)	0.07 (0.59)	0.06 (0.65)	0.09 (0.45)	0.13 (0.64)
R-meas	0.095	0.073	0.071	0.109	0.158
CC1/2	0.99 (0.97)	1.0 (0.96)	0.99 (0.84)	0.99 (0.97)	0.99 (0.94)
R_work_/R_free_	0.174/0.217	0.139/0.158	0.216/0.249	0.197/0.236)	0.185/0.222
Number of non-hydrogen atoms (Protein/ligands/water)	5979/12/844	962/4/144	3728/9/256	3683/10/26	1092/1/123
RMSD bonds (Å)	0.008	0.008	0.008	0.008	0.009
RMSD angles (°)	1.13	1.22	1.17	1.09	1.26
Ramachandran favored/allowed/outliers (%)	98/1.87/0.13	100/0/0	99/1/0	98/2/0	95/5/0
Average B-factor (Å^2^)	25.1	20.7	43.7	62.2	26.0

^*^Values in parentheses are for the highest-resolution shell.

^#^Values after anisotropic correction.

**Table 2:** Crystallographic data of GG-SpaA mutant proteins*.

	E139A	E269A	D295N	D295N/E269A
Beamline	BM14-ESRF	BM14-ESRF	BM14-ESRF	BM14-ESRF
Wavelength (Å)	0.95372	0.95372	0.95372	0.97876
Resolution range (Å)	50.0–2.47 (2.55–2.47)	50.0–2.39 (2.47–2.39)	50.0–2.6 (2.69–2.60)	50.0–1.99 (2.1–1.99)
Space group	*C* 2	*C* 2	*C* 2	*C* 2
Unit cell constants a, b, c (Å); β (°)	223.95, 63.49, 104.45; 94.99	228.63, 63.03, 104.91; 95.34	164.33, 75.16, 176.48; 100.3	229.7, 64.39, 105.05
No. of Unique reflections	44441 (3861)#	59092 (5823)	64377 (6215)	103663 (15000)
Multiplicity	5.0 (4.0)	5.1 (5.1)	14.4 (3.6)	4.1 (4.1)
Completeness (%)	83.53 (27.02)#	99.18 (98.78)	98.68 (96.19)	99.3 (99.0)
Mean I/σ (I)	17.36 (3.84)	16.00 (2.14)	14.43 (3.35)	12.6 (2.4)
Wilson B-factor (Å^2^)	14.05	45.89	46.07	29.25
R-merge	0.08 (0.35)	0.06 (0.61)	0.05 (0.27)	0.07 (0.64)
R-meas	0.099	0.083	0.072	0.089
CC1/2	0.99 (0.97)	0.99 (0.95)	0.99 (0.96)	0.99 (0.89)
R_work_/R_free_	0.201/0.217	0.219/0.237	0.242/0.265	0.205/0.228
Number of non-hydrogen atoms (Protein/ligands/water)	6049/38/286	6036/0/171	11668/0/244	5965/0/619
RMSD bonds (Å)	0.009	0.009	0.014	0.014
RMSD angles (°)	1.10	1.19	1.48	1.47
Ramachandran favored/allowed/outliers (%)	97/3/0	98/2/0	98/2/0	97/3/0
Average B-factor (Å^2^)	24.4	62.4	64.70	46.9

^*^Values in parentheses are for the highest-resolution shell.

^#^Values after anisotropic correction.
