# Supplementary material for: New insights about pilus formation in gut-adapted Lactobacillus rhamnosus GG from the crystal structure of the SpaA backbone-pilin subunit
Source: Sci Rep. 2016 Jun 28;6:28664. doi: 10.1038/srep28664 (PMC4923907; doi:10.1038/srep28664)
Supplement: Supplementary Information [file srep28664-s1.pdf]

Supplementary Information:

New insights about pilus formation in gut-adapted *Lactobacillus rhamnosus* GG from the crystal structure of the SpaA backbone-pilin subunit

Priyanka Chaurasia[1], Shivendra Pratap[1], Ingemar von Ossowski[2], Airi Palva[2] and Vengadesan Krishnan[1*]

[1] Regional Centre for Biotechnology, NCR Biotech Science Cluster, Faridabad – 121 001, India
[2] Department of Veterinary Biosciences, University of Helsinki, Helsinki, Finland

[*] Correspondence: kvengadesan@rcb.res.in

This document includes supplementary Figures (S1-S4) and Tables (S1 and S2).

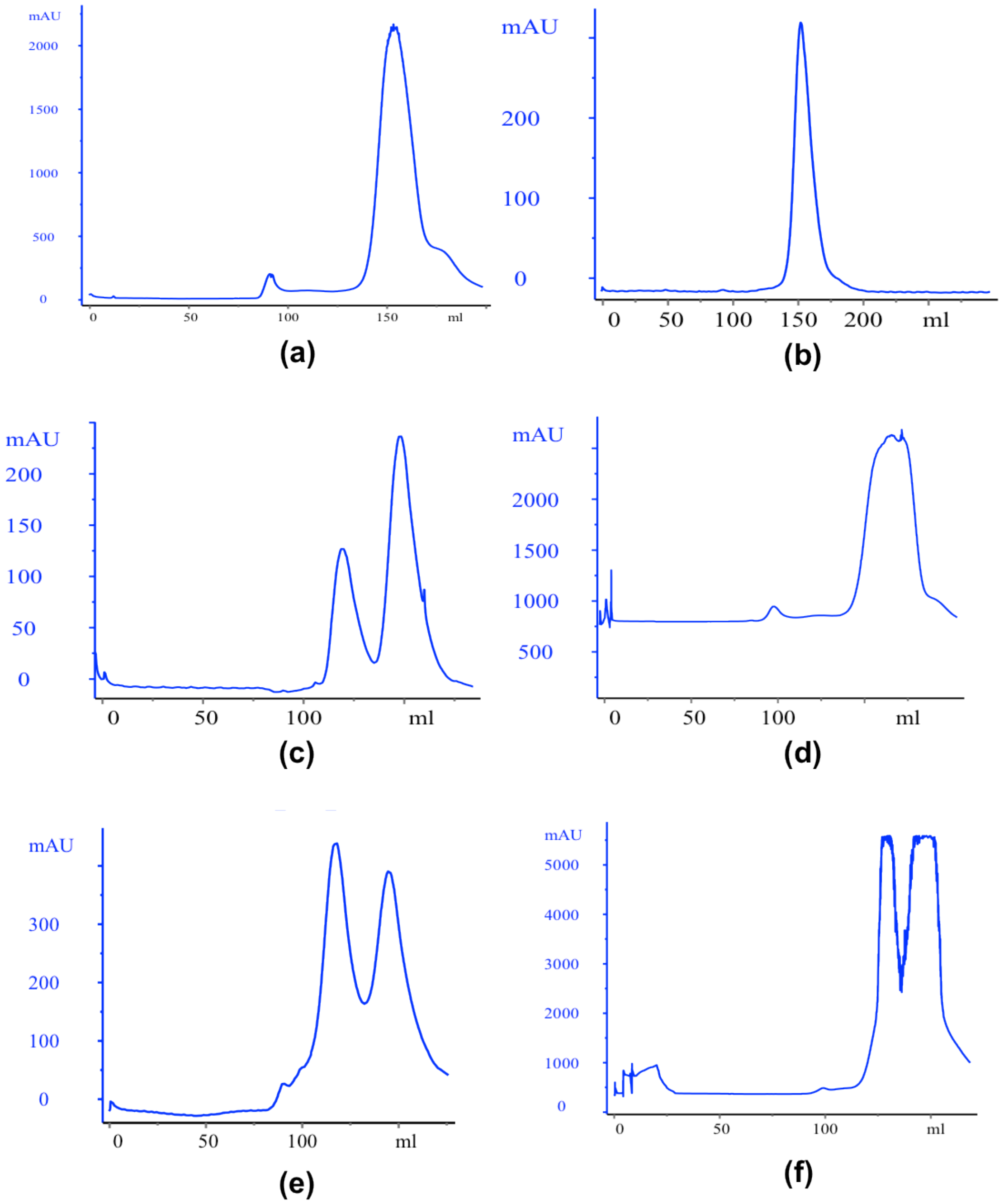

Figure S1. Size exclusion profile of purified WT and mutant GG-SpaA proteins. A single peak (E2) of monomers is eluted at 150 ml for the **(a)** WT, **(b)** E139A, and **(d)** D295N proteins. An additional peak (E1; trimeric protein) is eluted at 120 ml for the **(c)** E269A, **(e)** E139A/E269A, and **(f)** D295N/E269A proteins. The values on *x*- and *y*-axes are elution volume (ml) and UV (A280) absorbance (mAU), respectively.

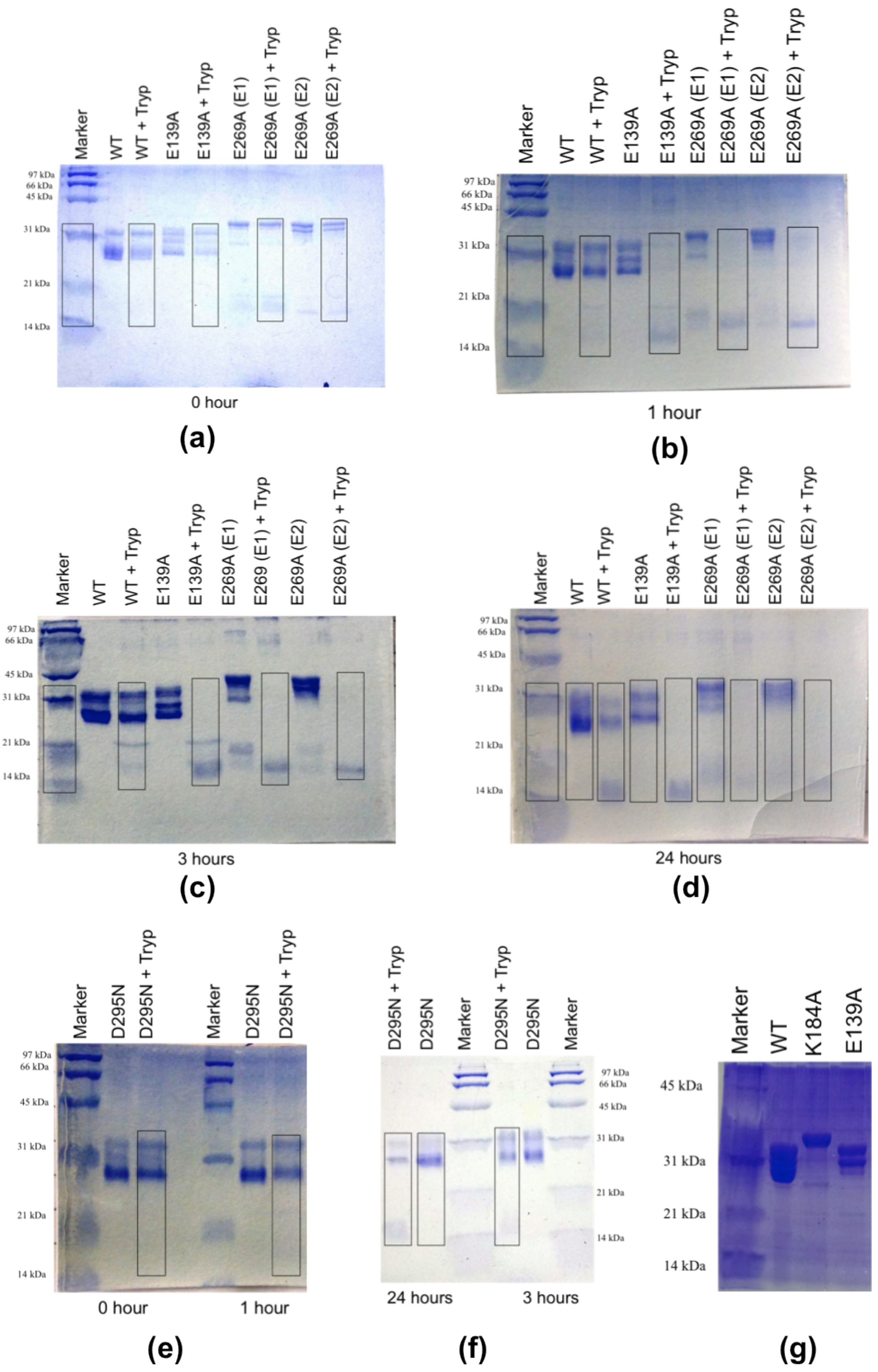

Figure S2. SDS-PAGE analysis of WT and mutant GG-SpaA proteins.
(a-f) Trypsinized GG-SpaA proteins (WT and the E139A, D295N, E269A (E1) and E269A (E2) mutants) were sampled at various time points (0, 1, 3, and 24 h) and analyzed by SDS-PAGE. Molecular weight markers (kDa) and labels for each lane are indicated at the side and top of the gels, respectively. Cropped regions used Figure 5A are outlined by boxes (solid lines). **(g)** A coomassie-stained gel that shows the electrophoretic mobility of WT and mutant (K184A and E139A) GG-SpaA proteins. Molecular weight markers (kDa) are shown.

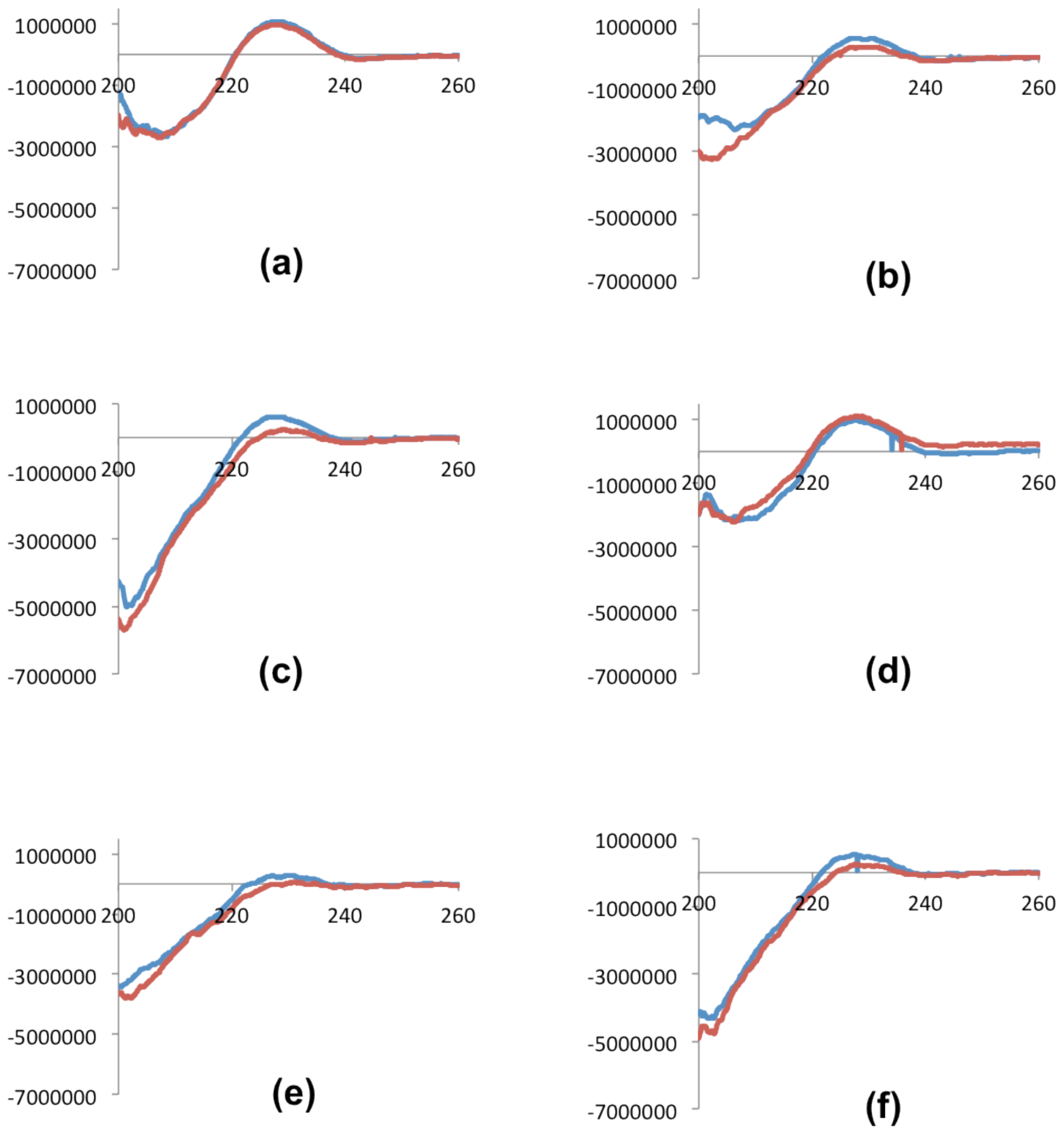

Figure S3. Far-UV circular dichroism (CD) spectra of WT and mutant GG-SpaA proteins. Thermal unfolding and refolding of GG-SpaA proteins was assessed by CD spectral analysis: **(a)** WT, **(b)** E139A, **(c)** E269A, **(d)** D295A, **(e)** E139A/E269A, and **(f)** D295N/E269A. Measurements (blue line) were taken initially at room temperature (20°C) and then after being heated (95°C) and cooled down again to room temperature (red line). Plotted on the *x*- and *y*-axes are wavelength (nm) and mean residue ellipticity, respectively.

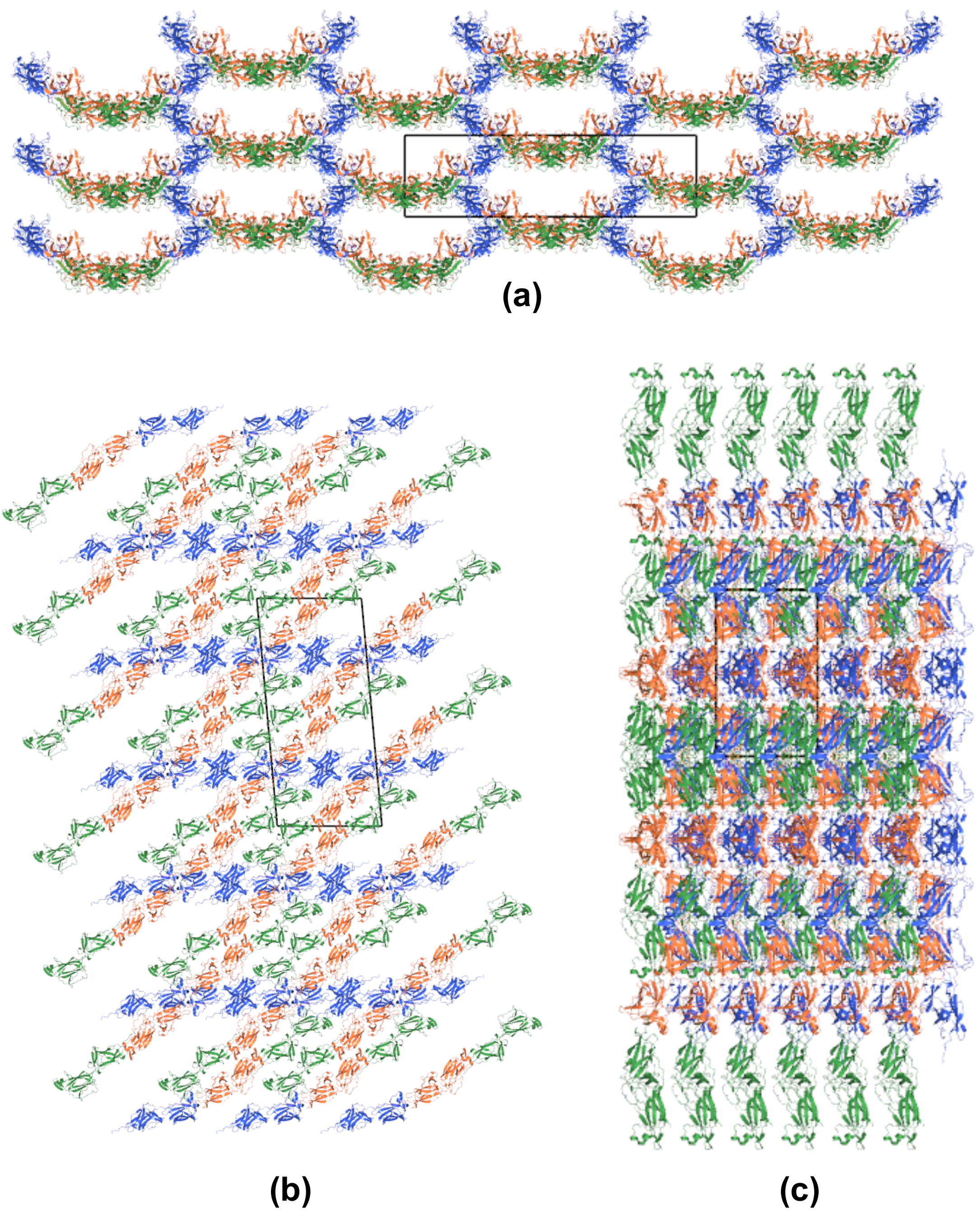

Figure S4. Molecular packing of the WT GG-SpaA crystal. Shown are representative views of the crystal packing along z-axis **(a),** y-axis **(b),** and x-axis **(c)**. The three molecules in the asymmetric units are colored red (chain A), blue (chain B), or green (chain C). Sold black lines outline the unit-cell edges.

Table S1. DALI PDB search for structural homologs of GG-SpaA*.

PDB code	Name	Pilin type	Host source	Z-score	RMSD (Å)	No. of aligned residues	Sequence identity (%)
**Full-length:**							
4UZG	BP-2b	backbone	*S. agalactiae*	14.6	5.3	130	25
2XTL	BP-2a	backbone	*S. agalactiae*	11.9	2.3	112	28
4HSS	SpaD	backbone	*C. diphtheriae*	11.9	5.6	143	20
4OQ1	RrgC	tip	*S. pneumoniae*	11.6	4.8	148	16
2Y1V	RrgB	backbone	*S. pneumoniae*	11.1	2.5	114	25
**N-domain:**							
4HSS	SpaD	backbone	*C. diphtheriae*	11.9	2.8	116	22
3PHS	GBS52	basal	*S. agalactiae*	10.2	2.6	107	18
4P0D	T6	backbone	*S. pyogenes*	10.2	2.1	93	20
3UXF	FimP	backbone	*A. oris*	10.1	2.4	108	25
3KPT	BcpA	backbone	*B. cereus*	9.6	2.5	92	21
**C-domain:**							
4UZG	BP-2b	backbone	*S. agalactiae*	14.7	1.5	103	27
3PHS	GBS52	basal	*S. agalactiae*	12.0	1.7	97	18
2XTL	BP-2a	backbone	*S. agalactiae*	11.8	2.3	110	27
4OQ1	RrgC	tip	*S. pneumoniae*	11.5	2.3	101	18
2Y1V	RrgB	backbone	*S. pneumoniae*	11.3	2.5	112	26

*Top five hits are listed.

Table S2. Oligoprimers used for site-directed mutagenesis of GG-SpaA.

Name	Nucleotide sequence*
E139A:forward	5'-GCTGTCTATCTTTTCCAT**GCA**ACCAATCCGCGAGCTGGT
E139A:reverse	5'-ACCAGCTCGCGGATTGGT**TGC**ATGGAAAAGATAGACAGC
E269A:forward	5'-ACAACTTACACGGCAGTT**GCA**ACAAACGTGCCGGATGGT
E269A:reverse	5'-ACCATCCGGCACGTTTGT**TGC**AACTGCCGTGTAAGTTGT
D295N:forward	5'-TCGTCTAGCGACATTCTA**AAT**GCACCAAGCGGTATTCTG
D295N:reverse	5'-CAGAATACCGCTTGGTGC**ATT**TAGAATGTCGCTAGACGA
K184A:forward	5'-TATGAGCGCACTTTTGTT**GCG**AAAGATGCTGAGACTAAA
K184A:reverse	5'-TTTAGTCTCAGCATCTTT**CGC**AACAAAAGTGCGCTCATA

*Mutated codons are in red font
